# Adapting to Uncertainty: A Mixed-Method Study on the Effects of the COVID-19 Pandemic on Expectant and Postpartum Women and Men

**DOI:** 10.3389/fpsyg.2021.688340

**Published:** 2021-11-24

**Authors:** Inês M. Tavares, Joana Fernandes, Catarina V. Moura, Pedro J. Nobre, Mariana L. Carrito

**Affiliations:** Faculty of Psychology and Educational Sciences, Center for Psychology at the University of Porto, Porto, Portugal

**Keywords:** pregnancy, postpartum, stress, anxiety, depression, couple relationship, COVID-19

## Abstract

Detrimental biopsychosocial effects of the COVID-19 pandemic on populations have been established worldwide. Despite research indicating that the transition to parenthood is a vulnerable period for maternal and paternal health, an in-depth examination of the specific challenges the pandemic poses for new mothers and fathers is still lacking. Using a mixed-method design, we investigated individual and relational well-being of women and men who were expecting their first child during the first months of the COVID-19 pandemic in Portugal and its associations with contextual, individual, and relational factors. Adults older than 18 (*n* = 316, 198 women) from early pregnancy to 6-months postpartum completed a cross-sectional online survey assessing sociodemographic, individual (depression, anxiety, perceived stress), and relational (dyadic adjustment, perceived social support) self-report measures. From those, 99 participants (64 women) responded to an open-ended question and reported perceived changes in their couple’s relationship due to the pandemic. Men responding during strict lockdown measures reported significantly higher levels of perceived stress relative to those men who were not under lockdown. Overall, women reported higher levels of depression and greater social support than men. Qualitative analyses resulted in two main themes: Individual Changes and Relational Changes. These themes aggregate personal concerns and experiences (e.g., worsening of mental health, uncertainty about the future, lack of freedom) interrelated with relational issues (e.g., increased togetherness, avoidance of physical contact, and increased availability for parenthood during lockdown). The prevalence of negative effects (58.6%) exceeded the described positive effects (28.3%), and 13.1% described both positive and negative effects of the pandemic. Current findings offer grounds for important evidence-based strategies to mitigate the potential adverse effects of the current pandemic on new mothers’ and fathers’ individual and relational well-being.

## Introduction

*Covid-19 came up in my postpartum period, a difficult time of adaptation to a new reality and routine. COVID-19 intensified moments of stress and anxiety*, (…*) it also shattered some idealization of the moment. The worst is the constant fear of getting sick, both of us and our baby. All this affects our well-being and leads to a lack of [sexual] desire and patience.*

CD, female, 3-months postpartum

The coronavirus disease (COVID-19) has been declared a pandemic by the World Health Organization (WHO) on March 12, 2020. The COVID-19 pandemic is now considered a major long-term stressor and its biopsychosocial impacts have been widely established, including increased prevalence of depression and anxiety symptoms in various populations (Bendau et al., 2020; Luo et al., 2020; Pfefferbaum and North, 2020). Nevertheless, there are still a number of unknowns on the impact of COVID-19 on several particularly vulnerable populations. One of such cases refers to individuals transitioning to parenthood, which face one of the most demanding life periods. Pregnancy and postpartum impose several biopsychosocial changes that require individuals to adjust and increase the risk of experiencing psychological and relational problems (Vismara et al., 2016; Doss and Rhoades, 2017; Da Costa et al., 2019). Whether and how these novel demands experienced by new parents affect their individual and/or dyadic well-being depends on the interaction between individual, relational, and contextual factors (Lazarus and Folkman, 1984; Ben-Zur, 2019).

During the COVID-19 pandemic, pregnant women were specifically considered a vulnerable group and were recommended to take additional precautions (Di Mascio et al., 2020; Phoswa and Khaliq, 2020; Wastnedge et al., 2021). Many countries limited non-emergency health care services in order to diminish contact between health care workers and patients as well as to ensure that resources were effectively placed on COVID-19 care provision and, consequently, these measures affected antenatal and postnatal healthcare services. For instance, in Portugal, similarly to what happened in many other countries, many women experienced a reduction or suspension of antenatal and postnatal health care services as their routine consultations were either suspended or replaced with video or telephone consultations. These imposed changes may have caused additional stress to expectant and postpartum women and their partners during an already vulnerable life stage, amplifying the negative impact of the pandemic. Also, expectant women and men may suffer different consequences of the pandemic, as sex- and gender- specific factors are among the most important determinants of health and disease outcomes (Spagnolo et al., 2020) and, as recent studies have started to uncover, are also important determinants of the psychological and emotional effects of COVID-19 (van der Vegt and Kleinberg, 2020; Zamarro and Prados, 2021).

In response to the pandemic, many governments worldwide have imposed a series of confinement and physical distancing measures as an additional attempt to control the spread of the infection. While these measures are effective in preventing the spread of the disease, isolation measures also pose detrimental effects to the physical and mental health of the populations, including negative effects on psychological and physical health, cognitive functioning, individuals’ quality of life and, importantly, interpersonal relationships (Cornwell and Waite, 2009; Barger, 2013; Cacioppo et al., 2015). These physical isolation and social distancing (i.e., “stay at home”) measures have resulted in families and couples being confined together at home or, instead, physically isolated from one another to decrease the risk of virus transmission. A series of factors can emerge during and after social isolation periods that pose negative mental health consequences for the individual, including anxiety related to the pandemic and fear of infection (Bai et al., 2004; Desclaux et al., 2017), boredom and absence of social outlets outside the home (DiGiovanni et al., 2004; Cava et al., 2005), or financial insecurity (Mihashi et al., 2009; Jeong et al., 2016).

Although prior studies have focused on the psychological effects of quarantine on individuals, the novel and challenging dynamics induced by the current pandemic (e.g., balancing individual vs. shared time during confinement, novel work-related responsibilities including working from home) may also have critical implications for couples. For instance, a Global Times (2020) newspaper article documented a peak in divorce rates in some districts of Xi’an, the capital of Northwest China’s Shaanxi province, during March 2020, as an immediate consequence of the COVID-19 outbreak. Preliminary research findings also indicate that the COVID-19 pandemic and related measures are linked to increased relationship conflict and worse psychological well-being among partnered individuals, compared to before the pandemic (Luetke et al., 2020; Yang and Ma, 2020). Nonetheless, emerging evidence also reveals that, in the context of the COVID-19 crisis, both positive and negative repercussions to individuals and couples might occur (Günther-Bel et al., 2020). This mixed picture relates to the fact that, on the one hand, the imposed COVID-19 “stay at home” measures may facilitate conflict and relational distress as couple members experience a sudden disruption to their daily routines and readjust to work and recreational activities; navigate physical distancing/disinfection measures due to concerns of contagion; face financial concerns/job disruption; and spend most or all of their time together in a limited physical space. On the other hand, this proximity can be protective against negative outcomes such as loneliness (e.g., Lauder et al., 2004; Shiovitz-Ezra and Leitsch, 2010; Bruce et al., 2019) and can create opportunities for increased intimacy, closeness, and communal problem-solving. For pregnant and postpartum couples, this experience of proximity and increased time together might be particularly valuable as it can be an opportunity to be highly engaged and establish a deeper bond with their child, which ultimately is beneficial for both individuals’ as well as for the child’s well-being (Shin et al., 2008; Clowtis et al., 2016).

Whether the COVID-19 crisis, as an intense external stressor, might threaten or strengthen couples’ relationships can be better understood through the conceptual framework of the vulnerability-stress-adaptation model (Karney and Bradbury, 1995; Pietromonaco and Overall, 2020). This model suggests that COVID-19 crisis creates a series of related stressors (e.g., confinement, economic strain, job loss) that have the potential to interfere with the relationship by increasing negative processes within the couple (e.g., hostility, estrangement, less responsive support). The extent to which these effects will negatively impact the relationship depends on each partner’s individual vulnerabilities (e.g., anxiety, depression) and on preexisting stressors (e.g., having a lower income or going through a particularly challenging life period such as the postpartum). As such, the presence of greater preexisting contextual vulnerabilities coupled with individual vulnerabilities of one or both partners will exacerbate the impact of pandemic-related stressors. The way in which expectant and postpartum couples particularly perceive the impact of the COVID-19 pandemic, and whether these perceptions are associated with preexisting contextual and individual vulnerabilities, is still currently unexplored. Prior research has found that women and men may respond differently to crisis and stressful events (Spagnolo et al., 2020; van der Vegt and Kleinberg, 2020; Zamarro and Prados, 2021). Recent studies show that, during the COVID-19 pandemic, the burden for mothers increased and remained higher compared for fathers (Del Boca et al., 2020; Farré et al., 2020). Furthermore, women seem to be more worried about their family and friends and tend to report more severe health concerns, such as anxiety and fear, while men are greatly worried about economical and societal concerns (van der Vegt and Kleinberg, 2020). This is also in line with studies demonstrating an overall increased prevalence and severity of depressive, anxious, and posttraumatic symptoms in women in comparison to men, including during the COVID-19 pandemic (Hodes and Epperson, 2019; Liu et al., 2020).

The present study aims to investigate how the current pandemic affects the relationships of women and men at a particularly vulnerable life stage such as pregnancy and postpartum, whether there are significant gender differences in these experiences, and to identify which of these individuals may be most at risk for adverse consequences during the COVID-19 crisis. To our knowledge, no studies have employed qualitative or mixed-methods procedures to answer this research question. Using a mixed-methods approach, we specifically aimed to: (a) describe women’s and men’s individual (i.e., perceived stress, anxiety, depression) and relational well-being (i.e., dyadic adjustment, social support) using validated self-report measures; (b) describe via qualitative analysis the ways in which women and men felt their relationship with their partner was impacted as consequence of the COVID-19 pandemic; (c) identify contextual (i.e., age, lockdown status, obstetric status) and individual (i.e., anxiety, depression) correlates of perceived individual and relational changes due to the pandemic, with particular attention to variations across gender and stage of pregnancy/postpartum.

## Materials and Methods

### Participants

A total of 316 participants (198 women and 118 men) were recruited as part of a study on psychological well-being during the transition to parenthood, at regularly scheduled clinical appointments at one of the largest national maternity and child health outpatient units, as well as through social media platforms and completed an online survey during the COVID-19 period in Portugal, between March 27 and November 24, 2020. Eligibility criteria for participation were: (1) age over 18; (2) able to read and write in Portuguese; (3) in a committed romantic relationship with a partner for at least 6 months; and (4) self or partner currently pregnant with their first child or currently up to 6 months postpartum at the time of assessment. All participants resided in Portugal at the time of participation and ranged in age from 19 to 47 years (*M* = 31.0, *SD* = 5.16). Almost all participants were of Portuguese nationality (91.3%). The majority of participants was cohabiting with their partner (93.1%). One-hundred and twenty-two participants (38.6%) responded while Portugal was under strict lockdown measures. Sociodemographic characteristics of the sample are summarized in Table 1.

**TABLE 1:** Sociodemographic characteristics of participants (*N* = 316).

	Women (*n* = 198)	Men (*n* = 118)
Age, *M* years ± *SD* (min—max)	30.5 ± 4.94 (19—42)	31.9 ± 5.42 (21—47)
**Obstetric status, *n* (%)**		
First/second trimester	34 (17.2%)	64 (54.2%)
Third trimester	90 (45.5%)	44 (37.3%)
Postpartum	74 (37.4%)	10 (8.50%)
Planned pregnancy (yes) *n* (%)	151 (77.8%)	88 (74.6%)
Responded during strict lockdown (yes) *n* (%)	61 (30.8%)	61 (51.7%)
**Education level, *n* (%)**		
≤12 years	70 (35.4%)	68 (58.1%)
Bachelor’s degree	74 (37.4%)	26 (22.0%)
Master’s degree	48 (24.2%)	21 (17.8%)
Ph.D.	6 (3.00%)	2 (1.70%)
**Working situation, *n* (%)**		
Working from home	37 (18.7%)	35 (29.7%)
Unemployed due to Covid-19	25 (12.6%)	15 (12.7%)
Essential worker	7 (2.8%)	17 (6.8%)
Other (e.g., on leave)	129 (65.2%)	22 (18.6%)
**Relationship status, *n* (%)**		
Dating	63 (32.1%)	40 (34.8%)
Married or Civil Union	133 (67.9%)	75 (65.2%)
Relationship duration, *M* years ± *SD*	6.65 ± 4.42	6.87 ± 4.31
**Cohabiting (yes)**		
*n* (%)	116 (58.6%)	101 (85.6%)
*M* years ± *SD*	4.13 ± 3.94	3.62 ± 3.01

### Procedure

This study received approval from the Ethics Committee at the Faculty of Psychology and Educational Sciences of the University of Porto and at the Centro-Materno Infantil do Norte. Participants were administered an online self-reported survey to investigate women’s and men’s well-being in the context of the COVID-19 pandemic. Before beginning the online survey, all participants received and reviewed information about the purpose and procedures of the study, including assurance of confidentiality, and provided their informed consent before participation. Each participant was compensated with a 10€ gift card as part of the larger study and, after completion of the study, individuals received information on relevant psychological resources during the COVID-19 pandemic.

A mixed-method design was used for this research to examine the ways in which participants describe how COVID-19 influenced their individual and couple functioning. First, participants completed questions on sociodemographics (e.g., age, gender, obstetric status), current pregnancy/obstetric health, as well as a series of previously self-report instruments validated to the Portuguese population on individual and relationship well-being. Then, participants answered an open-ended question eliciting data for qualitative analysis in which they were given the freedom to express and describe perceived changes in their relationships since the beginning of the COVID-19 crisis (“*In your own words, please describe how the COVID-19 pandemic influenced your intimate/couple relationship?”)*.

### Measures

#### Demographics

An initial questionnaire was used to collect participants’ basic information such as age, gender, obstetric health questions (such as pregnancy weeks/timing of postpartum, and whether the pregnancy was planned). Moreover, the date of participation was used to estimate whether participants were responding under or after national strict lockdown measures.

#### Depression

The well-validated Edinburgh Postnatal Depression Scale (EPDS; Cox et al., 1987; Matthey et al., 2001) was used as a measure of depressive symptoms. This 10-item scale is a screening tool for depression designed to particularly target populations at pregnancy/postpartum and has been validated for use in women and men with good psychometric properties. Participants were asked to rate the frequency with which they experienced symptoms of depression in the last 7 days with higher scores reflecting a higher presence of depressive symptoms. The Portuguese version of this measure has shown good internal consistency (α = 0.85; Figueiredo et al., 2007). Internal consistency of the EPDS in the current study was good (α = 0.84, 0.82 for women and men, respectively).

#### Anxiety

The Anxiety Subscale of the Hospital Anxiety and Depression Scale (HADS; Zigmond and Snaith, 1983) was used as a measure of anxiety. This widely used and well-validated subscale is comprised of seven items assessing the presence of symptoms of anxiety during the previous week, with higher scores indicating a more severe presence of anxious symptoms. The HADS has been translated to Portuguese and validated for use in Portuguese samples with good internal consistency (α = 0.76; Pais-Ribeiro et al., 2007). In the present study, the scale showed good indices of internal consistency (α = 0.83, 0.82 for women and men, respectively).

#### Dyadic Adjustment

The well-validated and widely used Dyadic Adjustment Scale–Revised (DAS-R; Busby et al., 1995) was used as a measure of dyadic adjustment. The DAS-R includes a comprehensive evaluation of different dimensions of adjustment in the dyadic relationship with a partner using 14-items (e.g., “How often do you and your partner calmly discuss something?”). Higher scores reflect higher levels of adjustment. The DAS-R has been validated for the Portuguese population (Costa, 2012) and, in the current study, showed good internal consistency indices (α = 0.82, 0.78 for women and men, respectively).

#### Perceived Stress

The Perceived Stress Scale (PSS; Cohen et al., 1983) was used to assess the degree to which individuals perceive situations in their lives as stressful. The scale includes 14 items asking participants to rate the frequency with which they experienced a given situation or feeling (e.g., “How often have you found that you could not cope with all the things that you had to do?”) in the previous month. Higher scores indicate higher perceived stress. The Portuguese version of the PSS yielded good internal consistency (α = 0.87; Trigo et al., 2010). In our study, Cronbach’s alpha values indicated good internal consistency (α = 0.89 for both women and men).

#### Perceived Social Support

The Multidimensional Scale of Perceived Social Support (MSPSS; Zimet et al., 1988) was included to assess the perception of social support individuals receive from three sources, each corresponding to a subscale: family, friends, and significant other. This brief measure is composed of 12 items, with higher scores reflecting higher degrees of perceived social support. The Portuguese validation yielded Cronbach’s alpha values between 0.85 and 0.95 for all three subscales (Carvalho et al., 2011). In this study, the scale showed excellent internal consistency for all subscales (for women and men, respectively: Family, α = 0.96, 0.96; Friends, α = 0.96, 0.96; Significant Other, α = 0.93, 0.91).

### Data Analysis

Given the goal of the current study of providing an in-depth understanding of the effects of the COVID-19 pandemic on the well-being of women and men transitioning to parenthood, we employed a mixed-method approach by combining qualitative and quantitative forms of analysis. The mixed-method approach provides a more comprehensive and ultimately ecologically valid understanding of the research question than each method does when employed by itself (Creswell and Plano Clark, 2011). Thus, we used validated quantitative measurement methods to assess individual and relationship functioning, which were integrated with descriptive, qualitative evidence of individuals’ perceived “effects” of the pandemic, resulting from participants’ qualitative (written) descriptions of what had changed. The integration of both methodologies offers the opportunity to understand the complexity of the phenomenon in more depth (Teddlie and Tashakkori, 2012).

#### Qualitative Data Analysis

From our total sample of 316 participants, 217 provided written responses reporting no changes in their couple relationships as a consequence of COVID-19 (e.g., “Everything continues as usual”). After dropping these cases, we used Braun and Clarke’s (2006) method of thematic analysis to code participants’ descriptions of change. After familiarization with the dataset, the thematic analysis involved identifying interesting data features or codes, clustering codes and searching for potential themes, and, finally, naming, defining, and redefining the themes and subthemes according to the six steps proposed by Braun and Clarke (2006). To ensure and increment the research’s validity, two authors (JF and CM) individually coded the raw data in an ongoing consensual review process, and the full team reviewed emerging results to reach a final thematic configuration. During the process of thematic analysis we observed theoretical saturation, which is considered the point at which no additional data are being found that add significant information to the research question (e.g., generate new codes, subthemes, or themes; Guest et al., 2020). Following the principles of qualitative research, reaching theoretical saturation indicates that the sample size is adequate to respond to the research question.

#### Quantitative Data Analyses

First, we provide descriptive statistics for psychological (depression, anxiety, and perceived stress) and relationship well-being (dyadic adjustment and perceived social support) in the sample focusing on potential differences regarding lockdown status, gender, and obstetric status (early-mid pregnancy vs. late-pregnancy vs. postpartum) of the participants. Many of our participants responded to our study in the first months of the European COVID-19 pandemic situation, hence lockdown status was explored as a potential impactful variable on participants’ indices of individual and relational functioning. Given that the normality assumption for the residuals’ distributions was mostly not confirmed, analyses were performed considering non-parametric tests. Group comparisons were performed using Mann-Whitney tests (for two groups) and Kruskal-Wallis tests (for more than two groups), and Bonferroni corrections for multiple comparisons were applied. Next, and in accordance with mixed-research procedures, we coded the previously defined qualitative change themes (as described above) into dichotomous variables representing the presence or absence of each specific qualitative theme. These dichotomous variables representing presence/absence of the qualitative themes were then used to test their association with the relevant study variables, permitting us to identify contextual (i.e., gender, obstetric status) and individual (i.e., depression, perceived stress) correlates of the pandemic-related perceived changes through cross-tabulation analysis. For all analyses, a *P*-value less than 0.05 was considered statistically significant. All statistical analyses were performed using SPSS, v24.0.

## Results

Descriptive statistics and Spearman correlation coefficients among study variables are depicted in Table 2.

**TABLE 2:** Descriptives and Spearman correlation coefficients among the study variables.

	M	DP	1	2	3	4	5
EPDS	6.39	4.15	–				
HADS	4.95	3.49	0.77**	–			
DASR	53.58	7.36	–0.33**	–0.34**	–		
PSS	15.39	6.70	0.77**	0.74**	–0.44**	–	
MSPSS	5.31	0.80	–0.41**	–0.38**	0.37**	–0.46**	–

**Correlation is significant at the.01 level.

### Lockdown Impact

We examined whether participants reported different indices of individual and relational well-being depending on whether they responded during or after strict lockdown measures. Male participants reported significantly higher perceived stress if under lockdown measures (*Z* = –2.08, *p* = 0.038, η^2^ = 0.02), while no statistically significant difference was found for female participants during or after strict lockdown measures (*Z* = –0.91, *p* = 0.363 η^2^ = 0.04). No other differences were found for other individual and relational functioning indices (depression, anxiety, perceived social support, and dyadic adjustment; all *p*_*s*_ > 0.121).

### Differences in Individual and Relational Indices of Well-Being

#### Gender Differences

**FIGURE 1:**
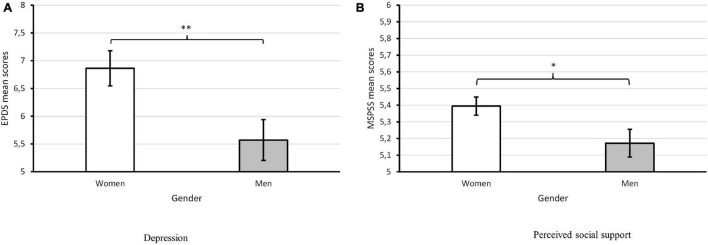
**(A**,**B)** Gender differences in depression (EPDS) and perceived social support (MSPSS). Error bars show standard errors of the mean. *p < 0.05, **p < 0.01.

Overall, women reported higher levels of depression (EPDS; *Z* = –2.82, *p* = 0.005, η^2^ = 0.03; see Figure 1A) and higher perceived social support (MSPSS; *Z* = –2.18, *p* = 0.034, η^2^ = 0.02; see Figure 1B) than men. More particularly, women reported greater perceived social support than men on the significant other (*p* = 0.032) and friends (*p* = 0.026) subscales, but no differences were found between women and men on the family subscale (*p* = 0.082). No other significant differences were found between women and men on the remaining individual (anxiety, perceived stress) or relational well-being indices (dyadic adjustment), as depicted in Table 3.

**TABLE 3:** Gender differences in depression (EPDS), anxiety (HADS), dyadic adjustment (DASR), perceived stress (PSS), and perceived social support (MSPSS).

	Women				Men				Z	*p*
	*n*	Mean	*SD*	Mean rank	*n*	Mean	*SD*	Mean rank		
EPDS	197	6.91	4.25	169.16	118	5.52	3.84	139.37	–2.82	0.005
HADS	197	5.12	3.49	162.21	117	4.67	3.48	149.57	–1.20	0.231
DAS-R	197	53.90	7.57	162.37	117	53.03	6.97	148.00	–1.36	0.174
PSS	183	15.63	6.60	151.66	112	15.00	6.86	142.03	–0.94	0.346
MSPSS	183	5.39	0.74	156.16	112	5.17	0.88	134.67	–2.20	0.034

#### Obstetric Status Differences

**FIGURE 2:**
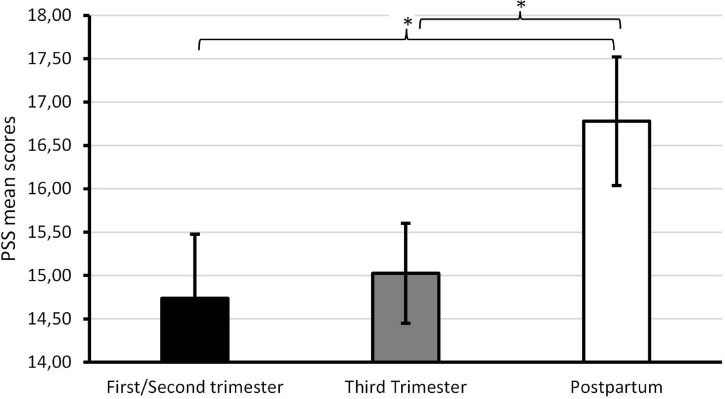
Perceived stress (PSS) scores at each moment of pregnancy/postpartum. *p < 0.05.

As can be seen in Table 4, stress, anxiety, and depression rates tended to be higher in participants who were at late pregnancy and at postpartum, while dyadic adjustment and perceived social support were slightly higher for third trimester participants, although these variations were not sufficient to flag significant differences between pregnancy/postpartum periods. Women and men at postpartum reported higher levels of perceived stress when compared to participants at early mid pregnancy (*p* = 0.039) and at late pregnancy (*p* = 0.046; see Figure 2).

**TABLE 4:** Individual and relational functioning indices according to obstetric status (pregnancy stage and postpartum).

	First/second trimester				Third trimester				Postpartum				H	*p*
	*n*	Mean	SD	Mean rank	*n*	Mean	SD	Mean rank	n	Mean	SD	Mean rank	*df* = *2*	
EPDS	98	6.10	4.24	150.40	134	6.46	4.04	160.90	83	6.63	4.24	162.29	1.02	0.604
HADS	98	4.77	3.74	149.64	133	4.87	3.50	155.38	83	5.30	3.17	170.17	2.45	0.294
DASR	98	53.04	7.09	148.71	132	54.49	6.65	167.20	83	52.76	8.56	150.57	2.93	0.232
PSS	92	14.74	7.06	138.95	126	15.02	6.48	143.00	77	16.78	6.50	167.00	5.30	0.071
MSPSS	92	5.25	0.82	140.26	126	5.36	0.82	154.73	77	5.31	0.74	146.24	1.60	0.448

**FIGURE 3:**
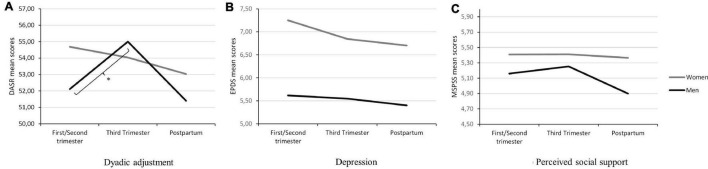
**(A–C)** Dyadic adjustment (DAS), depression (EPDS), and perceived social support (MSPSS) scores for women and men at each moment of pregnancy/postpartum. *p < 0.05.

When analyzing women and men separately, results indicated that, for men, dyadic adjustment was different according to the obstetric stage they were at [H(2) = 6.51, *p* = 0.039, η^2^ = 0.04], such that dyadic adjustment was higher for those men whose partner’s pregnancy was at the third trimester compared to those whose partner was at early mid pregnancy (*p* = 0.013; see Figure 3A).

Women did not show significant differences regarding their indicators of individual and relational functioning according to the moment of pregnancy or postpartum they were at (all *p* > 0.157; see Figure 3).

### Perceived Changes in Couples’ Relationship Due to the COVID-19 Pandemic

Concerning qualitative data, 99 participants described that COVID-19 had a marked impact on their intimate relationships. Compared to those who did not (*n* = 216), they differed in terms of their individual and relationship functioning in such a way that they reported higher depression (*Z* = –2.90, *p* = 0.004, η^2^ = 0.03), anxiety (*Z* = –3,39, *p* = 0.001, η^2^ = 0.04), and perceived stress rates (*Z* = –2.94, *p* = 0.003, η^2^ = 0.06), while they scored lower on dyadic adjustment (*Z* = –2.14, *p* = 0.033, η^2^ = 0.02) and perceived social support (*Z* = –2.73, *p* = 0.006, η^2^ = 0.05).

Out of those participants who indicated that the COVID-19 pandemic affected their relationships, most participants (*n* = 58, 58.6%) reported a negative impact, followed by 28.3% (*n* = 28) who described a positive impact and by 13.1% (*n* = 13) who considered that the pandemic exerted both a positive as well as a negative impact on their relationships. Three participants assumed that COVID-19 affected their couples’ relationship, but they did not describe how (e.g., “It had a positive influence”). For this reason, they were not included in the final thematic analysis (*n*_*final*_ = 96; 62 women and 34 men).

Two main themes emerged from the qualitative analysis of expectant and postpartum participants’ descriptions of changes in their intimate relationships due to the COVID-19 pandemic: *Individual Changes* and *Relational Changes*. These two main themes comprised 4 subthemes and 15 codes. Table 5 includes a complete description of the specific themes, component codes, and illustrative quotes for each. Interestingly, 31.2% of participants reported changes pertaining to the *Individual Changes* theme, 36.5% reported changes pertaining to the *Relational Changes* theme, and 32.3% reported changes pertaining to both themes.

**TABLE 5:** Results of the qualitative thematic analysis (*N* = 96).

Themes	Subthemes	Codes	Representative quotations
Individual changes	Psychological well-being	Restfulness	*Because we are at home for longer, the level of personal and emotional fatigue and wear is significantly lower. So, there is more propensity for intimacy* (LS, male, first trimester).
	Psychological distress	Global negative emotional state	*The current situation contains several implications that cause stress, anxiety, and concern about the future, which carries a negative psychological burden that affects all levels of our lives* (GM, male, third trimester).
		Fear of contagion	*The worst is the constant fear of being infected, both of us and our baby* (CD, female, 3-months postpartum).
		Health/care concerns	*The pandemic has affected us because we are concerned about our well-being and our families* (MS, female, third trimester).
		Uncertainty about the future	*It has a negative influence because it increased sadness and uncertainty about the future* (TS, female, third trimester).
		Economic/job concerns	*I am concerned about not being able to pay my bills. I can become unemployed without money to buy all the things for my baby and support my wife financially and psychologically* (RS, male, first trimester).
		Isolation/Confinement	*It negatively influenced everything in our lives since we were isolated without physical contact for 2 months* (LA, female, second trimester).
		Lack of freedom	*We don’t have the freedom to do what we like most* (BS, male, first trimester).
Relational changes	Dyadic adjustment	Togetherness	*There is more openness, empathy, and commitment to take care of each other and ourselves. There is also more peace and harmony. It was positive, strengthening closeness and affection* (AP, female, second trimester).
		Perceived enclosure	*Privacy in spaces is reduced* (PR, male, second trimester). *It did not allow us to miss our partner* (AM, female, third trimester).
		Tension and emotional weariness	*We are isolated at home for a long time, which resulted in emotional tension/wear sometimes* (TS, female, third trimester).
		Parenthood	*We are closer and enjoying pregnancy. I think it helped to bond with the baby* (JO, male, third trimester).
	Sexual adjustment	Avoidance of physical contact	*Because my partner is still working, the baby and I try to keep distance from my partner when he is at home* (JR, female, 3-months postpartum). *Avoidance of kisses and hugs* (MS, female, third trimester).
		Shift in priorities/Sex as secondary	*Stress, anxiety, and covid related concerns distanced us from thoughts about our sexual life [*…*] Decrease of sexual desire* (AG, male, third trimester).
		Increased availability for sex	*We spend more time at home together and are more available for intimate/sexual relationship* (DF, male, second trimester).

#### Individual Changes

The central theme **Individual Changes** aggregates responses that identified changes resulting from COVID-19 at an individual level. Many participants clearly differentiated detrimental effects on their personal mental health that were due to pandemic-related stressors (subtheme *Psychological Distress*). Out of the 96 responses, 58 participants (38 women and 20 men) described the experience of psychological distress (60.4%)—generally characterized by the individuals as a negative psychological burden, such as stress and anxiety—and experienced as a direct consequence of several interrelated worries and concerns that emerged during the pandemic.

Participants explained that the COVID-19 pandemic elicited a set of worries that increased their own levels of anxiety, stress, and sadness (see codes in Table 5). This distressful psychological experience was described by most individuals as having an effect on several areas of their lives (e.g., AC, female, 3-months postpartum, “*The stress that resulted from the pandemic has affected us in all sectors of our lives*”). The pandemic-related worries leading to individual psychological distress included general uncertainty about the future, health-related or care-related concerns (e.g., worries about nobody being available to take care of vulnerable family members, lack of child or maternal care), and unsettling concerns regarding economic strain and job loss (e.g., IF, female, third trimester, “*It caused anxiety related to uncertainty about work and our daughter’s safety during birth*”). Being afraid of infection (own or relatives), being isolated, and lacking freedom were also described as causes of individual psychological distress (e.g., HR, male, third trimester, “*I am always afraid of getting infected without knowing it and transmitting it to my wife*”).

Despite the psychological consequences of this challenging situation, two participants (2.1%) perceived an improvement in their emotional well-being (subtheme *Psychological well-being*). For these participants, being confined at home with their partner was experienced as an opportunity to relax and increase enjoyable activities with their partners, which ended up promoting their individual as well as their relational well-being. Importantly, participants also consistently described their levels of psychological functioning during the pandemic (whether deteriorated or increased) as a central determinant of the experienced changes in their relationships with their partners and linked to particular changes to their dyadic and sexual adjustment.

#### Relational Changes

Changes in the couples’ relationship associated with quarantine and COVID-19 restrictions were described in the second main theme **Relational Changes.** Sixty-six participants (68.8%; 44 women and 22 men) reported this theme. Some participants focused their responses on the changes experienced in their romantic relationship with their partner in a broad sense (*n* = 33, 34.4%, subtheme *Dyadic Adjustment*) or on the sexual changes more particularly (*n* = 26, 27.1%, subtheme *Sexual Adjustment*), while others described changes in both of them (*n* = 7, 7.3%).

Out of those participants who perceived changes to their dyadic relationship (*n* = 40; 33 who indicated dyadic changes and 7 who indicated both changes on sexual and dyadic adjustment), most of them referred that the COVID-19 pandemic exerted positive impacts (*n* = 21, 52.5%). Participants noted that, as a consequence of the confinement measures, they enjoyed more free time with their partner and the baby, because quarantine created opportunities for increased closeness and fostered deeper personal relationships (e.g., JO, male, third trimester, *“We are closer now and enjoying pregnancy. I think it has helped us to bond with the baby”*). Notwithstanding, seven participants (17.5%) also noted negative changes to their relationships, describing that spending all of their time together with their partner created conditions for conflicts and emotional weariness, leading to a possible estrangement (e.g., ES, female, 3-months postpartum, “*We spend all of our time at home and sometimes we argue*”).

For sexual adjustment, however, some participants (*n* = 23, 69.7%) reported a decrease in the frequency of sexual contact, desire, and availability for sex due to the pandemic. The COVID-19, as a challenging situation, prompted a change in individuals’ current priorities, and made sexual contact less of a priority (e.g., JL, female, third trimester, “*The anxiety that resulted from the pandemic changed my mood and caused anger and demotivation in general, namely in my sexual life”*). Fear of infection was also noted as a reason that led participants to avoid physical/intimate contact. In contrast, some participants (*n* = 7, 21.2%) indicated an increase in availability for sex. Spending and enjoying time together during their confinement period at home also created opportunities for improvement in their sexual lives (e.g., DF, male, second trimester, “*We spend more time at home together, and we are more available for our intimate/sexual relationship*”).

### Correlates of Perceived Individual and Relational Changes

**FIGURE 4:**
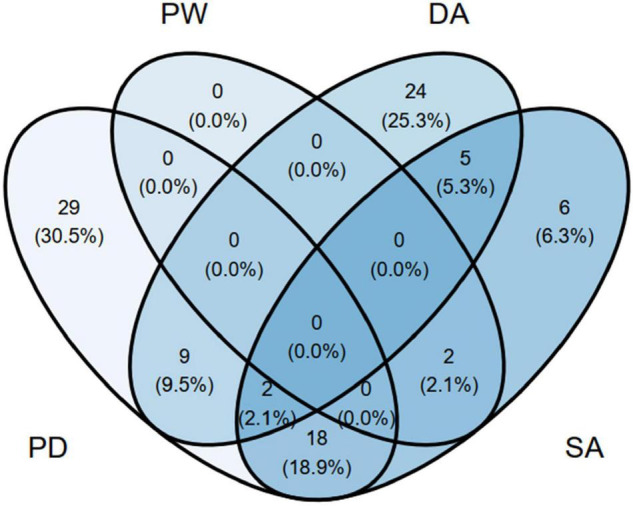
Percentages of participants’ overlap according to their description of each main subtheme (*n* = 96). PW = Psychological well-being subtheme; PD, Psychological distress subtheme; DA, dyadic adjustment subtheme; SA, Sexual adjustment subtheme.

As is typical of thematic analysis, one individual’s description could comprise several distinct codes pertaining to distinct subthemes and, as such, the overlap between the subthemes of the thematic analysis is represented in Figure 4. Binomial tests examining whether there were significant differences between individuals in the likelihood of reporting particular themes and subthemes indicated that the proportion of reported individual (*p* = 0.010) and relational (*p* < 0.001) themes was different from chance (1-sided). Regarding the subthemes, the same assumption was true for Psychological Well-being (*p* < 0.001) and Sexual Adjustment (*p* = 0.003), while marginally significant results were found for the Psychological Distress subtheme (*p* = 0.052). The proportion of reported Dyadic Adjustment subtheme was not different from chance (*p* = 0.125). Women did not differ from men on whether they reported each specific theme or subtheme (all *p*_*s*_ > 0.098). Likewise, themes and subthemes were not likely to be different for participants with different obstetric status (all *p*_*s*_ > 0.106).

The following analyses explored the link between participants’ self-reported characteristics and the reported themes and subthemes. As for sociodemographic and contextual factors, chi-square tests indicated that participants who reported that their pregnancy was not planned were more likely to describe *Relational changes* (with 89.5% reporting this theme) than participants with a planned pregnancy (with 63.6% reporting this theme, *p* = 0.03). No differences were found regarding educational level, household income, occupation, and pregnancy risk. As for individual correlates, participants who described Psychological Distress reported higher perceived stress scores (PSS) than participants who did not describe this subtheme (*Z* = –2.19, *p* = 0.028, η^2^ = 0.05). The remaining indicators of individual and relational functioning (EPDS, DAS-R, MSPSS) were not significant predictors of theme or subtheme selection (*p*_*s*_ > 0.082).

## Discussion

The current study was aimed at providing a comprehensive examination of women’s and men’s perceptions of the impact of the COVID-19 pandemic on their intimate relationships, as well as to identify key contextual, individual, and relational aspects relevant to the experience of these experiences of change. Employing a mixed-method approach that combines both quantitative and qualitative methodologies, and with theoretical insights from models on how couples adjust to intense external stressors (e.g., Karney and Bradbury, 1995; Pietromonaco and Overall, 2020), the results of the current study indicated two main interrelated dimensions of change: *Individual Changes* and *Relational Changes*. Transversely to the stage of pregnancy or postpartum they were at, both women and men alike identified particular dimensions of improvement and decline in their individual and relational well-being as a result of the current pandemic. Particular contextual and individual factors (e.g., unplanned pregnancy, greater perceived stress) were significantly linked to negative pandemic-related experiences. These findings provide additional insights on the well-being of individuals who transition to parenthood during the COVID-19 crisis, which are relevant for both clinical and research purposes.

Current COVID-related stressors, compounded with the specific challenges of pregnancy and postpartum, may increase women’s and men’s vulnerability for negative individual and relational outcomes during an already particularly challenging period for many (Moyer et al., 2020; Ostacoli et al., 2020; Salehi et al., 2020). The current study indicated that, as expected, the impact of the pandemic was not homogenous, with two thirds (69%) of all participants explicitly describing no changes to their intimate lives as a result from the pandemic. This finding is in line with recent research indicating that the effects of the pandemic do not have a marked effect on most individuals and/or couples (Panzeri et al., 2020) and reinforces that a set of factors might protect these couples from these detrimental effects. Comparing to those who identified significant changes in their relationships due to COVID-19, we found that those who did not report such alterations consistently reported higher levels of both individual and relational functioning (i.e., higher levels of depression, anxiety, stress, and lower rates of dyadic adjustment and perceived social support). For those women and men whose relationships were affected due to the current pandemic crisis, our results show a heterogenous pattern of changes. The majority of these male and female participants (58.6%) described negative changes due to the current pandemic, with only 28.3% of participants noting positive changes in their relationships. Interestingly, 13.1% of new parents reported both positive and negative effects. This finding is in accordance with recent studies demonstrating that COVID related stressors might concurrently induce an improvement and a deterioration in several indices of individual’s and couple’s well-being (Günther-Bel et al., 2020). Indeed, this mixed picture is distinctly translated in the findings of our qualitative analyses, in which participants refer to experiences such as increased conflict and emotional tension due to the lack of privacy and the constant time together at home, while also noting an improvement in their “togetherness” and availability for intimate and sexual interactions, describing the pandemic as a valuable opportunity to connect and be more present for their newborn baby. The observed pattern of positive, negative, and mixed descriptions of change reinforces the complexity and variability of quarantine ramifications for couples and families (Panzeri et al., 2020; Pietromonaco and Overall, 2020).

The thematic analysis of participants’ descriptions of change due to COVID-19 revealed two major interconnected themes: *Individual Changes* and *Relational Changes*. While a third (31.2%) of women and men reported only individual changes, another third (36.5%) reported only relational changes, and another third (32.3%) of participants described both individual and relational changes due to the pandemic. These findings show that both individual and relationship challenges were triggered by COVID-19, shaping mental health and relationship dynamics. Regarding the first theme, *Individual Changes*, these were closely connected to alterations to own’s mental health as a consequence of COVID-19. Most participants who reported these changes considered the current pandemic as a psychologically distressing period accompanied by negative feelings such as stress, anxiety, and depression (cf. code *Global Negative Emotional State*). These negative feelings were well connected to *health and care concerns* regarding the baby, the partner, other family members, and themselves, decreased perceived safety and stability regarding their jobs and finances (cf. code *Economic/Job Concerns)*, increased general *uncertainty about the future*, constant *fear of contagion*, as well as experiences of *isolation* and *lack of freedom* due to the social isolation and confinement measures. Altogether, these interrelated but distinct concerns and worries that emerged during the pandemic for most expectant and postpartum women and men led to personal psychological distress and difficulties adjusting to the current challenges. This is consistent with various studies that have reported predominantly negative psychological effects during the pandemic, including in similar samples (Brooks et al., 2020; Ostacoli et al., 2020).

The described experience of psychological distress had an indirect effect on participants’ interactions with their romantic partner. Particularly, women and men considered that their own fear of contamination and the restrictive measures put in place to prevent COVID-19 infection importantly contributed to relationship difficulties (cf. codes *Avoidance of Physical Contact, Perceived Enclosure*). The intense COVID-related concerns and own negative feelings (cf. *Psychological Distress* subtheme, as previously described) were identified as precursors of dyadic and sexual problems (cf. codes *Tension and Emotional Weariness, Shift in Priorities/Sex as Secondary*). In other words, as individuals focus and time were increasingly dedicated to pandemic-related stressors, this ultimately imposed negative effects on their romantic and sexual relationship. Indeed, previous literature focusing on the outcomes of quarantine/confinement measures, even in contexts other than the COVID-19 pandemic, indicates that the personal psychological difficulties due to these measures also pose extended, deleterious effects on their interpersonal and sexual relationships (Cacioppo et al., 2015; Günther-Bel et al., 2020; Panzeri et al., 2020). Notwithstanding, a small number of participants revealed positive changes in individual functioning as a consequence of having more free time and a more harmonious, restful life due to the current pandemic (cf. code *Restfulness*). In these cases, and as opposed to those in which participants reported increased psychological distress (e.g., anxiety, stress), participants adjusted positively to COVID-19 stressors and reported lower levels of fatigue and decreased emotional wear. For these participants, staying at home created opportunities to be more available to their relationship with their partner and with the baby (cf. codes *Togetherness*, *Parenthood*, *Increased Availability for Sex*).

The second major theme, *Relational Changes*, comprises a series of relationship alterations (cf. subtheme *Dyadic Adjustment*) and sexual-related alterations (cf. subtheme *Sexual Adjustment*) resultant from the COVID-19 pandemic. Men and women identified the emergence of novel relationship processes, such as increased communication and openness between partners, commitment, and empathy, which were frequently reported in the current study (cf. code *Togetherness)*. For these participants, stay-at-home measures increased their couple-focused time, which influenced their availability, energy, and interest in sexual activity, ultimately resulting in positive dyadic and sexual experiences. Such findings align with prior evidence showing an improvement in couples’ dynamics and increased sexual frequency during the pandemic (Günther-Bel et al., 2020; Yuksel and Ozgor, 2020). The transition to parenthood is a challenging and stressful life transition *per se* and some of these women and men had to face their daily life locked at home without the help of friends or family or with no life distractors (cf. code *Perceived Enclosure*). Novel couple dynamics that emerged because of COVID-19 demands were considered a source of relationship conflict among some participants (cf. codes *Tension and Emotional Weariness*). These individuals experienced low relationship cohesion and high relationship tension, which hampered their dyadic functioning and placed sexual activity as less of a priority due to the emerging conflicts and decreased sexual desire (cf. Code *Shift in Priorities/Sex as Secondary*), congruently with what has been reported in recent studies (Luetke et al., 2020; Panzeri et al., 2020). Sexual changes in their relationship due to the pandemic were identified by the majority of participants (cf. subtheme *Sexual Adjustment*) and included complaints on decreased sexual motivation, sexual desire, and sexual frequency (cf. code *Shift in Priorities/Sex as Secondary*). These women and men considered the pandemic as a predisposing factor for the emergent sexual difficulties, by eliciting fear of contamination during intimate and sexual interactions, by being a mentally exhausting time, or by placing on them several other competing priorities and concerns in their lives. Besides, some of these participants shared their homes with other family members, resulting in perceived lack of privacy and fewer moments of intimacy with the partner. Overall, new parents’ perspectives on their sexual lives during the pandemic show support to previous pandemic evidence by indicating that, for some, there might be a decrease of sexual behaviors, while others experience an increase in sexual connection and intimacy (Jacob et al., 2020; Luetke et al., 2020).

Altogether, the identified dimensions of change put forward a heterogeneous and multidimensional description of the impacts of COVID-related stressors, confinement and social isolation measures, changes to maternal healthcare during the pandemic, as well as the particular dyadic and parenting challenges that arose for new parent couples. In effect, an interesting and novel finding of our study is the observed overlap between particular dimensions of change in new parents’ individual and relational lives. For instance, changes in couples’ sexual adjustment were particularly interconnected with experiences of personal distress (18.9% of participants concomitantly reported both subthemes), rather than with experiences of personal well-being (2.1% of participants concomitantly reported both subthemes). A central question of the current study was to identify which particular subsets of individuals might be at heightened risk for the potential deleterious effects of the pandemic. We identified several correlates of poorer intrapersonal and interpersonal functioning. First, both women and men who were at postpartum showed greater levels of perceived stress than those who were at pregnancy during the pandemic. Whereas women demonstrated overall higher levels of depression and increased social support than men, an increased level of stress was found for men, but not women, who were under lockdown measures. Increased stress postpartum is associated with decreased sensitivity to and engagement with a newborn’s cues (Shin et al., 2008; Clowtis et al., 2016) and to mothers’ and fathers’ postpartum depression (Vismara et al., 2016; Da Costa et al., 2019). Stress has also been found to hinder couples’ relationship functioning and longevity (Randall and Bodenmann, 2017). Given these detrimental effects, it is critical that future research and clinical efforts consider key aspects such as stress management strategies for men under lockdown (e.g., duration of confinement, efforts to maintain social support while socially distanced) and postpartum experiences for women and men (e.g., stress regulation strategies during postpartum).

Conversely, we also identified factors which were associated with better levels of individual and relational functioning for women and men. When connecting their contextual and individual characteristics and their verbal written descriptions of COVID-19 effects, we found that those participants with a planned pregnancy were less likely to describe relational changes (with 63.6% reporting this theme vs. 89.5% of those with an unplanned pregnancy reporting this theme), underscoring the effects of an unintended pregnancy on the well-being of women and couples (Barton et al., 2017). Unsurprisingly, for those who described pandemic-related psychological distressful outcomes, perceived stress was significantly higher than for those who did not experience such outcomes. Given the negative effects of stress on important physical and psychological indices of health (Larzelere and Jones, 2008), and considering the particular challenging character of the transition to parenthood, our study’s findings offer grounds for evidence-based strategies to mitigate the potential adverse effects of stress related to the current crisis on individual and relational well-being. Effective strategies to manage stress during and after lockdown, to sustain social support, and to better navigate postpartum-specific challenges during COVID-19 may help new mothers and fathers to successfully maintain their individual and relationship well-being during the current pandemic.

This study contributes to a much-needed area of research during the current pandemic, but its findings should be considered in light of some limitations. Although the study is sustained in prior theoretical and empirical research (e.g., Karney and Bradbury, 1995; Pietromonaco and Overall, 2020), this study was correlational and did not follow individuals over time as the pandemic unfolded. Future longitudinal studies should explore the temporal associations of the observed findings. Data were collected online, which limited participation to couples with access to online resources and might have prevented us from capturing deep responses. These would be possible using interviews instead of discrete items but using interviews would be less suitable in the context of a pandemic, possibly increasing non-compliance. Also, the current study mirrors new mothers’ and fathers’ personal perspectives but does not inform on interdependency of perspectives with participants’ respective partners. All individuals who participated in this study were in intimate, mixed-sex relationships, and were transitioning to parenthood for the first-time. It is unknown whether results generalize to more diverse samples or to those who are faced with additional stressors (e.g., same-sex couples, adoptive parents, parents to an infant born preterm) and this might be explored in future research. Despite these limitations, this study offers a novel, comprehensive perspective on the impact of COVID-19 on expectant and postpartum women and men. Resulting from the integration of both quantitative and qualitative results, the current findings can guide researchers and clinicians in targeting the specific challenges which have emerged during the pandemic for these individuals and to the development of effective strategies to promote new mothers’ and fathers’ well-being.

## Data Availability Statement

The raw data supporting the conclusions of this article are available upon reasonable request from the corresponding author IMT.

## Ethics Statement

The studies involving human participants were reviewed and approved by the Ethics Committee at the Faculty of Psychology and Educational Sciences of the University of Porto and at the Centro Materno-Infantil do Norte. The patients/participants provided their written informed consent to participate in this study.

## Author Contributions

IT, JF, and MC: conceptualization. IT, JF, CM, and MC: methodology and writing—original draft. IT, JF, PN, and MC: writing—review, and editing. IT, PN, and MC: funding acquisition. PN and MC: supervision. All authors contributed to the article and approved the submitted version.

## Conflict of Interest

The authors declare that the research was conducted in the absence of any commercial or financial relationships that could be construed as a potential conflict of interest.

## Publisher’s Note

All claims expressed in this article are solely those of the authors and do not necessarily represent those of their affiliated organizations, or those of the publisher, the editors and the reviewers. Any product that may be evaluated in this article, or claim that may be made by its manufacturer, is not guaranteed or endorsed by the publisher.
